# Cx43 Promotes Endothelial Cell Migration and Angiogenesis via the Tyrosine Phosphatase SHP-2

**DOI:** 10.3390/ijms23010294

**Published:** 2021-12-28

**Authors:** Hanna Mannell, Petra Kameritsch, Heike Beck, Alexander Pfeifer, Ulrich Pohl, Kristin Pogoda

**Affiliations:** 1 Walter Brendel Centre of Experimental Medicine, Munich University Hospital, Ludwig-Maximilians-Universität München, Großhaderner Str. 9, 82152 Planegg-Martinsried, Germany; hanna.mannell@med.uni-muenchen.de (H.M.); kameritsch@lmu.de (P.K.); heike.beck@med.uni-muenchen.de (H.B.); upohl@lmu.de (U.P.); 2 Clinical Pharmacy, University Hospital, Ludwig-Maximilians-Universität München, Marchioninistraße 15, 81377 München, Germany; 3 Institute of Pharmacology and Toxicology, Biomedical Center University of Bonn, Sigmund-Freud-Straße 25, 53105 Bonn, Germany; alexander.pfeifer@uni-bonn.de; 4 Physiology, Institute for Theoretical Medicine, University of Augsburg, Universitätsstraße 2, 86159 Augsburg, Germany

**Keywords:** connexin, Cx43, tyrosine phosphatase, SHP-2, migration, angiogenesis

## Abstract

The gap junction protein connexin 43 (Cx43) is associated with increased cell migration and to related changes of the actin cytoskeleton, which is mediated via its C-terminal cytoplasmic tail and is independent of its channel function. Cx43 has been shown to possess an angiogenic potential, however, the role of Cx43 in endothelial cell migration has not yet been investigated. Here, we found that the knock-down of Cx43 by siRNA in human microvascular endothelial cells (HMEC) reduces migration, as assessed by a wound assay in vitro and impaired aortic vessel sprouting ex vivo. Immunoprecipitation of Cx43 revealed an interaction with the tyrosine phosphatase SHP-2, which enhanced its phosphatase activity, as observed in Cx43 expressing HeLa cells compared to cells treated with an empty vector. Interestingly, the expression of a dominant negative substrate trapping mutant SHP-2 (CS) in HMEC, via lentiviral transduction, also impaired endothelial migration to a similar extent as Cx43 siRNA compared to SHP-2 WT. Moreover, the reduction in endothelial migration upon Cx43 siRNA could not be rescued by the introduction of a constitutively active SHP-2 construct (EA). Our data demonstrate that Cx43 and SHP-2 mediate endothelial cell migration, revealing a novel interaction between Cx43 and SHP-2, which is essential for this process.

## 1. Introduction

The growth of new blood vessels starts in existing vessels where new capillaries are sprouting through the proliferation and migration of endothelial cells. The activation of endothelial cells to proliferate and migrate is dependent on environmental signals like oxygen supply [1,2], the release of growth factors [3], and several signalling pathways like the Notch system expressed in vascular cells [4]. The connexin 43 (Cx43) has repeatedly been shown to be involved in angiogenesis [5,6,7,8,9].

Cx43 is a member of the connexin protein family, which are membrane proteins found in many different cells. From the 21 human connexins (Cx), Cx37, Cx40, Cx43, and Cx45 are expressed in vascular cells and—arranged in hexamers—form intercellular channels, the gap junctions [10]. They enable direct intercellular communication by allowing the exchange of molecules smaller than 1 kDa, thereby coordinating vascular function and communication [10]. In recent years, it has been repeatedly demonstrated that Cx additionally possess gap junction-independent functions [5,11,12,13,14]. The observed effect on angiogenesis via Cx43 seems to be part of its gap-junction independent function. For instance, ischemia dependent angiogenesis in the heart was shown to be unaffected by a gap-junction opener, but increased by Cx43 overexpression [8]. In addition, the formation of capillary like structures in vitro were shown to be affected by Cx43, but were independent of its gap-junctional communication [5]. Earlier studies have shown that the gap-junctional independent function of Cx43 is important for cell motility and migration [13,15,16]. Furthermore, direct interaction with several proteins involved in cell growth and death are associated with a channel independent effect of Cx43 [17]. These channel independent functions seem to be restricted to the C-terminus [10,17,18], which is located in the cytosol and is the main site of posttranslational changes [18,19], for instance phosphorylation, acetylation, or S-nitrosylation [18,20]. Phosphorylation can be observed at many serine, threonine, and tyrosine residues. Most of the phosphorylation sites have been associated with the regulation of the gap-junction channel activity [18,20,21,22], and their role in gap-junction independent processes still needs to be investigated at large. However, in a previous study, we showed that protein kinase A (PKA) phosphorylates C-terminal Ser 364, Ser 369, and Ser 373, and thereby regulates the gap junction independent migratory effect of Cx43 [16]. Next to kinases, phosphatases have been found to affect Cx43 function. Dephosphorylation of Cx43 by phosphatases PP-1 and PP-2A [20,23,24], as well as the T cell protein tyrosine phosphatase (TC-PTP) [25], affects gap-junctional communication. Whether phosphatases influence the channel independent functions of Cx43 has not yet been investigated. 

The Src-homology 2 domain containing tyrosine phosphatase SHP-2, a nonreceptor tyrosine phosphatase, is ubiquitously expressed [26,27] and is involved in many cellular functions [28,29,30,31]. Being involved in the regulation of cell proliferation, cell differentiation, and programmed cell death, SHP-2 additionally has an important role in cancer [32], as well as in the regulation of vascular signalling [33] and angiogenesis [34]. SHP-2 has repeatedly been shown to be important for endothelial cell signalling [31,34,35,36,37]. It promotes growth factor induced proliferation and angiogenesis in vitro and in vivo by activation of the PI3-K/Akt signalling pathway [34]. Furthermore, we showed that SHP-2 induces hypoxia dependent wound healing angiogenesis by promoting HIF-1α stabilisation and activation via Src and p38 MAPK dependent inhibition of the 26S proteasome [35,38]. Src dependent phosphorylation of Y247 and Y265 seems to be involved in the anti-proliferative effects of Cx43 [39], and growth inhibiting effects of Cx43 are reported to be channel independent [40]. However, whether tyrosine phosphorylations of Cx43 have an effect on the channel independent increase in cell migration is so far unknown. Interestingly, we previously demonstrated that SHP-2 binds to the C-terminal tail of endothelial connexin 37 (Cx37) and dephosphorylates the tyrosine residue at position 332 [41]. It has, however, not yet been investigated if SHP-2 interacts with Cx43 and if this plays a role in endothelial angiogenic responses, such as endothelial cell migration. 

Here, we investigated if Cx43 influences endothelial cell migration in vitro and vessel sprouting ex vivo. In addition, we studied the possible interaction between SHP-2 and Cx43, and its effect on endothelial migration.

## 2. Results

### 2.1. Downregulation of Cx43 Decreases Endothelial Cell Migration

**Figure 1:**
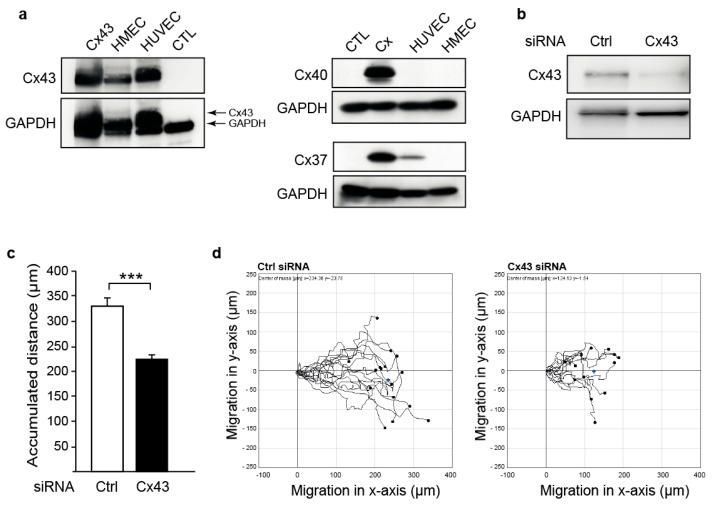
Downregulation of Cx43 reduces endothelial cell migration. (**a**) Western blot analysis of Cx43, Cx40, and Cx37 revealed that Cx43 is expressed in both human umbilical vein endothelial cells (HUVEC) and human microvascular endothelial cells (HMEC), whereas Cx40 and Cx37 are not expressed in HMEC. Cx43 (Cx43), Cx40 (Cx), and Cx37 (Cx) expressing HeLa cells were used as the positive controls. CTL: Wildtype HeLa cell lysate. (**b**) Western blot analysis of Cx43 in HMEC transfected with 100 nM siRNA against Cx43 or non-silencing control siRNA (Ctrl) for 48 h. The Cx43 expression was analysed using a polyclonal Cx43 antibody. Immunostaining for GAPDH was used as the loading control. (**c**) Serum-induced cell migration of HMEC transfected with a Cx43-siRNA or a control siRNA (Ctrl) in response to 10% FCS was assessed in a wound assay. Downregulation of Cx43 significantly decreased cell migration, represented as accumulated distance (*** *p* < 0.001 Ctrl-siRNA vs. Cx43-siRNA, *n* = 6, 3 independent cell cultures). (**d**) Representative single cell traces of migrated HMEC.

The migration of endothelial cells is one of the major processes during angiogenesis [42]. To investigate whether the migration of endothelial cells is affected by Cx43, we used human microvascular endothelial cells (HMEC), as these endogenously only express Cx43 (Figure 1a). Transfection with a specific siRNA (Cx43-siRNA) reduced Cx43 expression compared to a non-silencing control siRNA (Figure 1b). Downregulation of Cx43 significantly reduced cell migration of HMEC (accumulated distance in µm, mean ± SEM: Ctrl-siRNA: 324 ± 14; Cx43-siRNA: 238 ± 8; Figure 1c). Representative cell tracks are shown in Figure 1d. These results demonstrate that Cx43 positively affects cell migration in endothelial cells.

### 2.2. Downregulation of Cx43 Reduces Angiogenic Sprouting Ex Vivo

**Figure 2:**
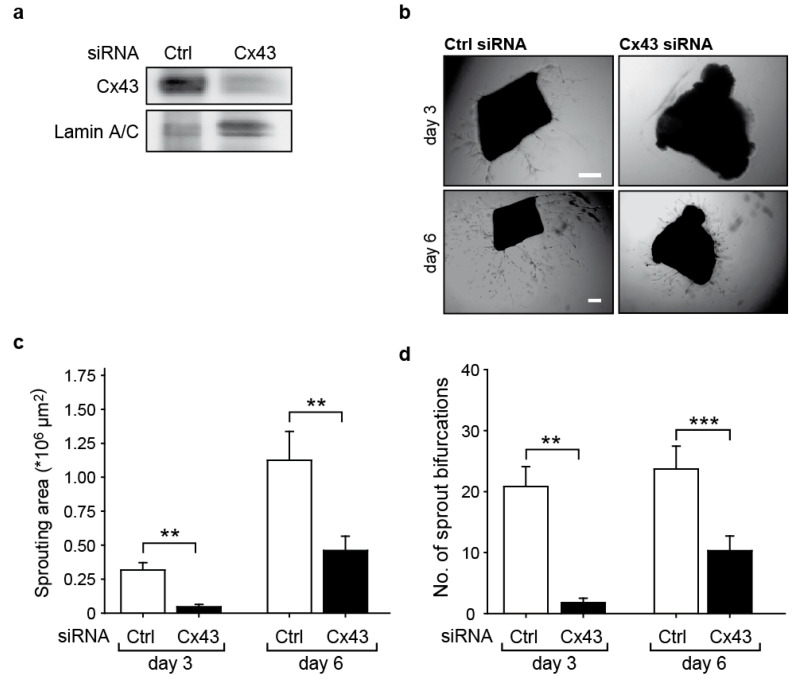
Downregulation of Cx43 impairs vessel sprouting ex vivo. (**a**) Downregulation of Cx43 in the mouse aortae was achieved by transfection with Cx43 siRNA (500 nM) or control (Ctrl) siRNA, as assessed by Western blot 48 h later. (**b**) Representative images of aortic sprouts in matrigel. Scale bar represents 200 µm. (**c**) The vessel sprouting area (** *p* < 0.01 Ctrl-siRNA vs. Cx43-siRNA, *n* = 2 aortae with eight aortic segments each at 3 days; *n* = 3–4 aortae with 6–8 aortic segments each at 6 days) and (**d**) the number of sprout bifurcations (** *p* < 0.01 Ctrl-siRNA vs. Cx43-siRNA, *n* = 2 aortae with 8 aortic segments each at 3 days; *** *p* < 0.001 Ctrl-siRNA vs. Cx43-siRNA *n* = 3–4 aortae with 6–8 aortic segments each at 6 days) were significantly reduced upon downregulation of Cx43, as assessed 3 days and 6 days after transfection in a matrigel assay.

To further investigate whether the effects on cell migration after Cx43 downregulation have functional consequences on angiogenesis, the sprouting of endothelial cells from isolated mouse aortae was assessed 3 days and 6 days after siRNA transfection. Aortic rings embedded in matrigel showed a significantly reduced new vessel sprouting after downregulation of Cx43 in the endothelium (Figure 2a). The sprouting area (Ctrl-siRNA: 1.12 ± 0.21 × 10^6^ µm; Cx43-siRNA: 4.60 ± 1.05 × 10^5^ µm; Figure 2c), as well as the number of bifurcations on sprouts (Ctrl-siRNA: 23.7 ± 3.7; Cx43-siRNA: 10.3 ± 2.8; Figure 2d), were significantly reduced upon downregulation of Cx43 compared to the treatment with control siRNA. Representative images of the aortic rings are shown in Figure 2b. 

### 2.3. Cx43 Interacts with the Tyrosine Phosphatase SHP-2 and Induces Its Activity

**Figure 3:**
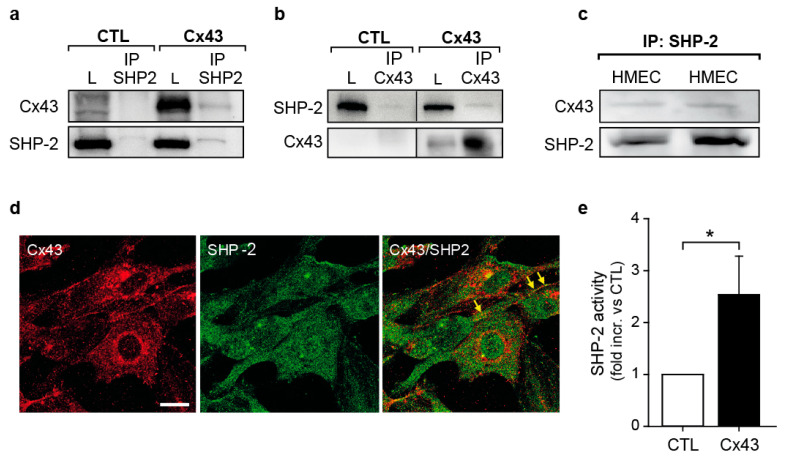
Interaction of Cx43 and SHP-2 induces SHP-2 phosphatase activity. (**a**) Upon immunoprecipitation (IP) of SHP-2 from HeLa cells expressing only Cx43, Cx43 was co-immunoprecipitated. As a control, cells transfected with an empty vector (control (CTL)) were used. L: Whole cell lysates. (**b**) SHP-2 was detected upon immunoblotting after immunoprecipitation of Cx43 from HeLa cells expressing only Cx43 in contrast to the control cells (CTL). L: Whole cell lysates. Images originates from the same blot, which was cropped; (**c**) SHP-2 was immunoprecipitated together with Cx43 in HMEC; (**d**) Co-localization of SHP-2 and Cx43 was detected by immunofluorescent staining of HMEC followed by confocal microscopic imaging. Scale bar represents 20 µm; (**e**) SHP-2 phosphatase activity was significantly increased in HeLa cells expressing Cx43 compared to HeLa cells transfected with an empty vector (* *p* < 0.05, *n* = 4 independent cell cultures), as assessed by detection of dephosphorylation of a phosphate analogue in SHP-2 immunoprecipitates.

SHP-2 is an important regulator of growth factor signaling during angiogenesis [33], and we have previously shown that Cx37 interacts with SHP-2 [41]. As the C-terminal part of Cx43 is phosphorylated at several tyrosine residues [19], we analyzed a potential interaction of SHP-2 and Cx43 in HeLa cells. As seen in Figure 3a, Cx43 could be detected in SHP-2 immunoprecipitates. This was confirmed by performing immunoprecipitation of Cx43 followed by the detection of SHP-2 (Figure 3b). In HMEC, an interaction of SHP-2 with Cx43 was also observed upon immunoprecipitation of SHP-2 (Figure 3c), as well as by immunofluorescent staining (Figure 3d). As the binding of SHP-2 to its targets induces phosphatase activity due to the release of the allosteric inhibition from its N-SH2 domain [27], we next measured the SHP-2 activity. The expression of Cx43 in HeLa cells significantly induced the phosphatase activity of SHP-2 compared to the cells lacking Cx43 expression (Figure 3e).

### 2.4. SHP-2 Inactivation Reduces Endothelial Migration

**Figure 4:**
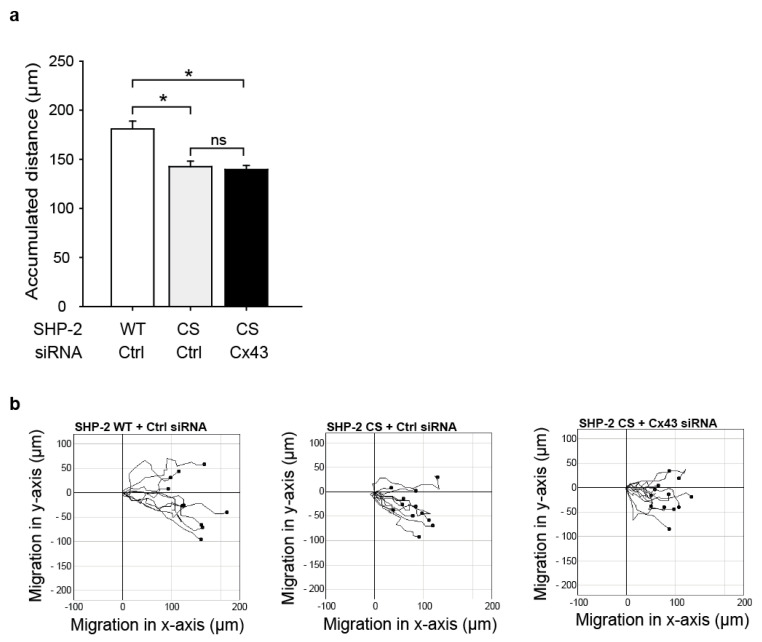
Inactivation of SHP-2 reduces endothelial cell migration. (**a**) Overexpression of a dominant negative substrate trapping mutant SHP-2 (SHP-2 CS) in HMEC significantly reduced serum (10%) induced endothelial cell migration, as assessed by the accumulated distance in µm in an in vitro wound assay (* *p* < 0.05, *n* = 3 independent cell cultures) compared to SHP-2 WT. Additional knock-down of Cx43 (Cx43 siRNA) did not further decrease migration (ns: not significant, *n* = 3 independent cell cultures). (**b**) Representative single cell traces of migrated HMEC.

As we observed an interaction between SHP-2 and Cx43, which induced the SHP-2 activity, we next investigated if SHP-2 influences the endothelial cell migration. As seen in Figure 4, the migration of HMEC was significantly reduced in the cells overexpressing a SHP-2 construct with a mutation in its catalytic pocket (Cys459 to Ser; CS), rendering the phosphatase inactive but with an intact phospho-binding ability (dominant negative substrate trapping mutant), compared to cells expressing the wildtype (WT) construct (accumulated distance in µm, mean ± SEM: WT: 181 ± 8; CS: 143 ± 6; Figure 4). Additional knock-down of Cx43 in SHP-2 CS overexpressing cells did not reduce the migration any further (accumulated distance in µm, mean ± SEM: Cx43-siRNA + SHP-2 CS: 140 ± 4; Figure 4).

### 2.5. The Impaired Migration in Cx43 Knock-Out Cells Cannot Be Rescued by Expression of a Constitutively Active SHP-2

**Figure 5:**
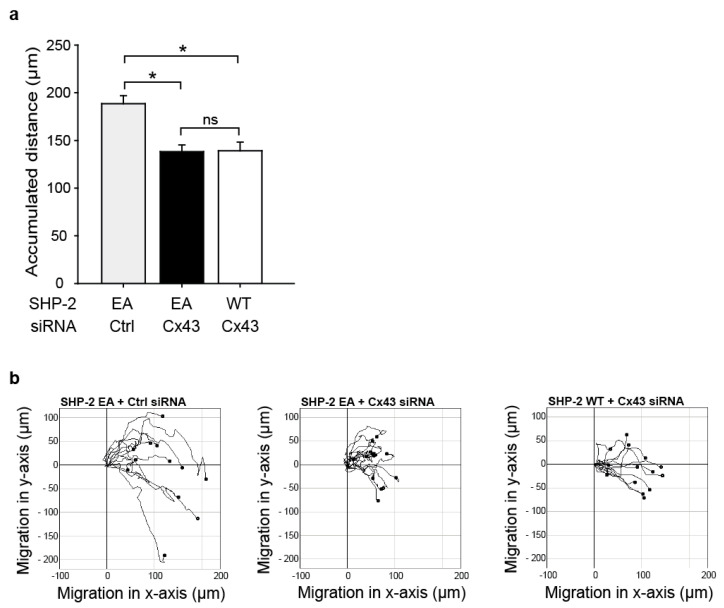
The Cx43 and SHP-2 interaction is vital for endothelial migration. (**a**) Treatment of HMEC overexpressing a constitutively active mutant SHP-2 (SHP-2 EA) with Cx43 siRNA did not rescue the reduced migratory response caused by Cx43 knock-down (Cx43 siRNA) (ns: not significant, *n* = 3 independent cell cultures) compared to SHP-2 EA cells treated with control siRNA (Ctrl) (* *p* < 0.05, *n* = 3 independent cell cultures), as assessed by the accumulated distance in µm in an in vitro wound assay (* *p* < 0.05, *n* = 3 independent cell cultures). (**b**) Representative single cell traces of migrated HMEC.

To study if the interaction between SHP-2 and Cx43 is essential for migration and if SHP-2 can promote migration via parallel pathways in endothelial cells, we overexpressed a constitutively active SHP-2 (Glu76 to Ala; EA) in HMEC in combination with Cx43 siRNA treatment. The overexpression of SHP-2 EA in Cx43 knock-out cells did not rescue the impaired migratory response (accumulated distance in µm, mean ± SEM: Cx43-siRNA and SHP-2 EA: 139 ± 7; Figure 5) compared to the expression of SHP-2 EA in the control cells (accumulated distance in µm, mean ± SEM: Ctrl-siRNA and SHP-2 EA: 189 ± 9). The treatment of cells expressing SHP-2 WT with Cx43 siRNA showed the same extent of reduction in migration as SHP-2 EA expressing cells treated with Cx43 siRNA (accumulated distance in µm, mean ± SEM: SHP-2 WT and Cx43 siRNA: 139 ± 9; Figure 5). This indicates that the SHP-2/Cx43 interaction is essential for the effect observed on endothelial migration.

## 3. Discussion

Cx43 has been shown to influence cellular responses, independently of its gap junctional channel function [5,11,12,13,14,39]. We previously showed that the Cx43 channel independent function is important for cellular migration [13,16]. Here, we show that Cx43 is involved in the angiogenic response of endothelial cells, and reveal the tyrosine phosphatase SHP-2 as a novel interaction partner of Cx43, which was essential for the Cx43 mediated endothelial migration.

In previous studies using HeLa cells expressing Cx43, we demonstrated that the C-terminus of Cx43 promotes cell motility and migration independently of gap junctional communication [13,16]. This promoting effect on migration was confirmed in endothelial progenitor cells by us [13] and others [6], but only indirectly in human endothelial cells, where a decoy peptide corresponding to the amino acids 363-374 in the C-terminal region of Cx43 containing the phosphorylation sites for PKA rescued the PKA dependent inhibition of endothelial migration [16]. Here, we used human microvascular endothelial cells, which only express Cx43, whereby possible interfering effects of Cx40 and Cx37 can be excluded. In HMEC, we demonstrated that the knock-down of Cx43 significantly reduced endothelial cell migration, strengthening and expanding our and others’ previous findings in other cell types. Moreover, we observed that the knock-down of Cx43 in the endothelium in mouse aortas impaired the aortic vessel sprouting ex vivo. This is in line with the recently published study by Koepple et al., where Cx43 siRNA treatment impaired the ability of endothelial cells to form capillary like structures in vitro [5]. Of note, Koepple et al. found the endothelial cell proliferation to be unaffected by Cx43, and therefore hypothesized that the angiogenesis promoting effect of Cx43 is derived from an influence on migration. Indeed, we found this to be true in our study. 

**Figure A1:**
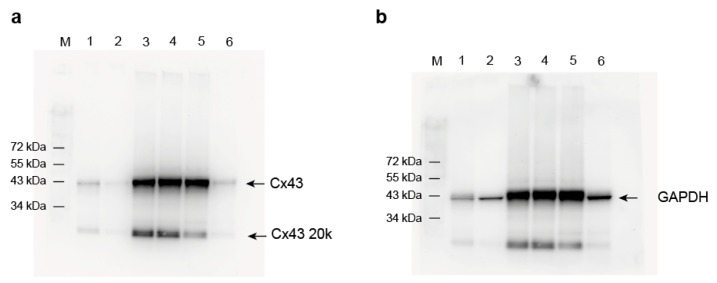
Whole size Western blots—detection of Cx43 after siRNA transfection. (**a**) Cx43 was detected after treatment with control siRNA and Cx43 siRNA, respectively. In addition, the short Cx43 isoform (GJA 1-20k; denoted as Cx43 20k in figure) was detected and shown to be downregulated by Cx43 siRNA. Lanes 1–2 are displayed in Figure 1b. (**b**) GAPDH was detected as a loading control. Traces of Cx43 protein bands can also be seen as the membrane was not stripped between antibody incubations. Lanes 1–2 are displayed in Figure 1b. M: Protein size marker.

The downstream signaling pathways responsible for the Cx43 dependent angiogenic effect remain to be investigated at large. Yu et al. found that Cx43 mediates HIF-1α and, subsequently, the VEGF expression under chronic cerebral hypoperfusion [7], whereas Wang et al. found a Cx43 dependent suppression of the c-jun N-terminal kinase (JNK) activity [9]. However, the involvement of these signaling pathways were not investigated in association with Cx43 mediated migration, and not much is known regarding the direct mechanism, and the involved proteins, of how Cx43 induces activation of angiogenic signaling. As a direct interaction partner in endothelial cells, the zonula occludens (ZO-1) was found [43]. This interaction was shown to be important for the link to the F-actin cytoskeleton and influenced cell spreading. Moreover, we found the p21-activated protein kinase 1 (PAK1) to directly bind to Cx43 and induce p38 MAPK activation, being responsible for filopodia formation and migration [13,15]. Furthermore, as mentioned before, we observed PKA mediated phosphorylation of Cx43 to inhibit cellular motility [16], demonstrating that inhibitory phosphorylations do not only exist in the context of the regulation of channel activity, but also for the gap junctional channel independent functions. Here, we also found indications that tyrosine phosphorylation is important for the Cx43 mediated migratory effect, as the tyrosine phosphatase SHP-2 was bound to Cx43 and this induced its phosphatase activity. Cx43 has been shown to become phosphorylated on tyrosine 265 in the carboxyl-tail by v-Src, thereby creating an SH2-domain binding motif, which enhances the binding of the v-Src SH2-domain to Cx43 [44]. It is therefore possible that SHP-2 binds to the Cx43 SH2-binding motif surrounding tyrosine 265 with one of its SH2-domains. Although SHP-2 contains two SH2-domains, and the engagement of both domains ensures maximal phosphatase activity due to a fully open state of the phosphatase domain, the occupation of only one SH2-domain leads to a semi active state, with increased activity compared to the closed state [45]. In addition, as Cx43 can be phosphorylated on other tyrosines [46], it is also possible that both SH2-domains are engaged. However, it still remains to be investigated if SHP-2 dephosphorylates Cx43 directly and thereby relieves an inhibitory mechanism or if the binding of Cx43 brings SHP-2 in close vicinity to other tyrosine phosphorylated targets important for cell migration. In addition, it remains to be investigated if SHP-2 also binds to the cytoplasmic 20kDa C-terminal fragment of Cx43 (GJA 1-20k) [47]. This short isoform of Cx43 is also expressed in HMEC and was additionally downregulated by Cx43 siRNA (Appendix A Figure A1). Hence, we cannot rule out that the Cx43 mediated migratory effects in endothelial cells are due to GJA 1-20k and not the full-length Cx43 or both, as we demonstrated in an earlier study that the expression of the C-terminus alone (amino acids 257–382) was sufficient to enhance migration [16]. Moreover, as the SH2-binding motif surrounding tyrosine 265 is also present in the 20kDa isoform, which starts at amino acid 213 [47], it is possible that SHP-2 binds to GJA 1-20k. However, this is an objective of our further studies. Nevertheless, as we observed a dephosphorylation of the tyrosine residue at position 332 in the C-terminal tail of Cx37 by SHP-2 in a previous study [41], it is likely that SHP-2 additionally dephosphorylates Cx43. Indeed, dephosphorylation of Cx43 by tyrosine phosphatases have been observed before. Li et al. found the T-cell protein phosphatase (TC-PTP) to dephosphorylate the Cx43 carboxyl-tail at positions Y247 and Y265, thereby counteracting channel closure [25]. However, the role of tyrosine phosphatases in the context of regulating the channel independent functions of Cx43 has not been investigated before. Importantly, we found the interaction of SHP-2 and Cx43 to play a significant role in endothelial cell migration. Overexpression of a substrate trapping mutant of SHP-2 lacking phosphatase activity significantly reduced endothelial migration, similar to Cx43 down-regulation. This is in accordance with previous results from us, where SHP-2 was shown to positively influence the growth factor and hypoxia induced angiogenesis in vitro and in vivo [34,35,38]. Interestingly, the diminished endothelial migration upon Cx43 knock-down could not be rescued by the introduction of a constitutively active SHP-2, demonstrating that the presence of Cx43 is essential for the SHP-2 mediated positive effect on endothelial migration. However, the exact underlying mechanisms and involved signalling proteins in this context still need to be investigated.

Taken together, we demonstrate that Cx43 and SHP-2 promote endothelial migration, and reveal that the novel interaction of Cx43 and SHP-2 is necessary for their migratory effects. This reveals an additional exciting mechanism of the possible regulation of the Cx43 gap junction channel independent activity.

## 4. Materials and Methods

### 4.1. Cells and Culture Conditions

HeLa cells stably transfected with the empty vector pBEHpac18 (HeLa-CTL) or stably expressing rat Cx43 (HeLa-Cx43) were a kind gift from Dr. Klaus Willecke (University of Bonn, Bonn, Germany). The cells were cultured in Dulbecco’s modified Eagle medium (DMEM, Invitrogen, Fisher Scientific, Schwerte, Germany) with 10% new born calf serum (NBCS, Biochrom, Merck, Darmstadt, Germany) and 1% penicillin/streptomycin, and were supplemented with 1 μg/mL puromycin (Sigma Aldrich, Merck, Darmstadt, Germany). Human microvascular endothelial cells (HMEC) were provided by Ades et al. [48] and were cultured in endothelial cell growth medium (DMEM supplemented with 10% fetal calf serum (FCS), 10% endothelial growth media (PromoCell, Heidelberg, Germany), and 1% penicillin/streptomycin (Sigma Aldrich)). All of the cells were maintained at 37 °C and 5% CO_2_.

### 4.2. Lentiviral Transduction of HMEC

Lentiviral vectors of WT SHP-2, the dominant negative substrate trapping SHP-2 CS (mutation of Cys459 to Ser) and the constitutively active SHP-2 E76A (mutation of Glu76 to Ala) were generated as previously described [35]. Lentiviral particles were produced and the biological titer was assessed by flow cytometry of transduced HEK293T cells, as described by Hofmann et al. [49]. The SHP-2 constructs were overexpressed in HMEC by lentiviral transduction, as previously described [35], at a multiplicity of infection (MOI) of 5. Shortly, HMEC were grown to 70–90% confluency in 60 mm dishes and were incubated with lentiviral particles diluted in Hank’s balanced salt solution for 4 h. Fresh growth medium was added, and the cells were cultured for 24 h before siRNA transfection.

### 4.3. siRNA-Mediated Knockdown

HMEC were transfected with synthetic siRNA (Qiagen, Hilden, Germany) to downregulate Cx43 (Cx43 siRNA) or with a non-silencing siRNA as the control (Ctrl). The cells were transfected with siRNA (100 nM) using a Hiperfect transfection reagent (Qiagen), according to the manufacturer’s instructions. The siRNA siCx43 was synthesized with the human Cx43 nucleotide sequences 5’-ATGCTTAGAGTGGACTATTAA-3´ as target (Qiagen). As a control, a non-silencing siRNA sequence (AATTCTCCGAACGTGTCACGT) was used (Qiagen).

### 4.4. Cell Migration 

Migration of HMEC was analyzed with wound assay chambers (Ibidi, Gräfelfing, Germany), as previously described [16]. Briefly, HMEC were transfected with 100 nM siRNA and after 48 h were detached with trypsin and were suspended in endothelial cell growth medium with 5% FCS. The two-well culture-inserts (Ibidi) were placed in eight-well µ-slide chambers (Ibidi) and were coated with collagen G (10 µg/mL in PBS, Biochrom) at 37 °C for 1 h. The cells were then seeded in each chamber of the 2-well culture-inserts (80 µL/chamber of 7 × 10^5^ cell/mL). The silicon inserts were removed after 24 h, and migration into the defined gaps was induced with endothelial growth medium containing 10% FCS. Migration was observed with a live cell imaging system (Ibidi) under an inverted microscope (Zeiss, Oberkochen, Germany) and the images were captured every 10 min for 12 h with an AxioCam camera (Zeiss). Migration was analyzed with the manual tracking software component in ImageJ and the plugin “Chemotaxis and Migration Tool” (Ibidi) to compute the accumulated distance (mean distance of all cell paths).

Migration of HMEC transduced with lentiviruses to overexpress SHP-2 WT, the dominant negative (SHP-2 CS) or active (SHP-2 E76A) SHP-2, and transfected with a control (Ctrl) or Cx43 siRNA for 48 h was followed for 8 h, as described above.

### 4.5. Mouse Aortic Ring Assay 

C57BL/6J wild type mice (Charles River, Burlington, MA, USA) were euthanized by cervical dislocation under anaesthesia (Midazolam 5 mg/kg; Medetomidin 0.5 mg/kg; Fentanyl 0.05 mg/kg) and the thoracal aortas were isolated. All of the animal studies were conducted in accordance with the German animal protection law. The aortas were catheterized at one end and mounted above a permanent magnet and flushed with serum-free DMEM to remove the blood rests. For siRNA transfections, the Effectene transfection reagent (Qiagen) was used in combination with magnetofection [50]. In detail, 500 nM siRNA was mixed with Enhancer (8 µL/µg siRNA), EC-Buffer, and 10 µg CombiMAG (Chemicell, Berlin, Germany), and was vortexed 5 s and incubated at room temperature for 3 min. Effectene transfection reagent was added to a final volume of 200 µL, followed by an incubation for 10 min at room temperature. The transfection solution (200 µL) was injected into the aorta via the catheter, followed by the application of the magnetic field for 30 min. Following magnetofection, the aortas were incubated in a humidified incubator with DMEM supplemented with 20% FCS for another 24 h. The aortas were cut into segments the next day, followed by embedment in growth factor reduced Matrigel (BD Biosciences, Heidelberg, Germany). The embedded aortas were then covered in DMEM supplemented with 20% FCS and 10% endothelial growth media, including endothelial growth factors (PromoCell), and were incubated for 6 days in total. Images of aortic rings were taken with a Zeiss Axiovert 200 M microscope at five-fold magnification. Angiogenesis was assessed by measuring the vessel sprouting area using the Zeiss AxioVision software and counting the number of sprout bifurcations. 

### 4.6. Western Blot Analysis

Western blot analysis was performed as previously described [16]. Briefly, cell lysates were prepared by scraping the cells in a Laemmli buffer [51]. The samples were boiled (5 min), size-separated by SDS-PAGE (8–16% Tris–Glycine gels, Thermo Fisher Scientific, Schwerte, Germany), and transferred electrophoretically to a Hybond-P membrane (Cytiva Amersham, Fisher Scientific). After blocking in 5% skimmed milk powder (AppliChem, Darmstadt, Germany) in PBS-0.1% Tween 20 (Sigma Aldrich) for 1 h, the membranes were incubated with primary antibodies overnight. Primary antibodies (anti-Cx43 (1:1000; Sigma Aldrich), anti-SHP-2 (Santa Cruz, Heidelberg, Germany), and anti-GAPDH (1:10,000; Merck) were diluted in 5% bovine serum albumin (BSA, AppliChem) in PBS-0.1% Tween. The membranes were washed with PBS-0.1% Tween and incubated for 2 h with the according secondary antibodies coupled to horseradish peroxidase (1:2000-1:5000; Merck) diluted in 5% skimmed milk powder in PBS-0.1% Tween. After washing, the bound antibodies were detected by enhanced chemiluminescence (ECL, Applichem). Detection of GAPDH was used to demonstrate equal loading. 

### 4.7. Lysis of Mouse Aortae

Then, 48 h after transfection, mouse aortae were covered with lysis buffer (cell signaling) supplemented with 100 µM PSMF and were shredded using ceramic beads (PeqLab, Erlangen, Germany) and shaking for 20 s in a CapMix (3M ESPE, Seefeld, Germany). Then, 30 µg of the supernatant was subjected to SDS-PAGE (10%), followed by Western blotting, as described before [52]. Lamin A/C (Santa Cruz) was used as an equal loading control.

### 4.8. Immunoprecipitation

HeLa cells were lysed in an ice cold lysis buffer (50 mM NaCl, 1% Triton X-100, 50 mM Tris–HCl pH 8.0) containing protease inhibitors (10 μg/mL aprotinin; AppliChem, 10 μg/mL leupeptin; AppliChem and 1 mM PMSF; Sigma Aldrich). Lysates were passed through a 26-gauge syringe needle to shear the genomic DNA and were centrifuged (15 min at 13,000 rpm, 4 °C). For immunoprecipitation, the supernatants were incubated over night at 4 °C with a mouse anti-SHP-2 antibody (Santa Cruz) or a rabbit anti-Cx43 antibody (Sigma Aldrich), and were magnetic beads coated with protein A (Cx43) or G (SHP-2) (Miltenyi Biotec, Bergisch Gladbach, Germany). Separation of the immune complex was performed with a magnetic separation unit (Miltenyi Biotec), according to the manufacturer´s instructions. After washing of the columns, the immunoprecipitates were eluted with a Laemmli buffer [51] and were analyzed for the binding of Cx43 or SHP-2 by Western Blot using a rabbit anti-Cx43 antibody (Sigma Aldrich) or a mouse anti-SHP-2 antibody (Santa Cruz). 

### 4.9. Immunofluorescent Staining

HMEC were grown on 8-well µ-slides (Ibidi, Gräfelfing, Germany) coated with 0.1% gelatine. Upon reaching 90–100% confluency, the cells were washed in a phosphate buffered saline supplemented with Ca^2+^ (PBS+) and fixated in 4% formalin for 10 min at room temperature (RT), followed by permeabilization with 0.1% triton X-100 (Sigma Aldrich) in PBS+ for 2 min at RT. After washing three times with PBS+, the cells were blocked with a blocking buffer (1% bovine serum albumin in PBS+) for 1 h at RT during gentle shaking, followed by incubation with primary antibodies (1:200 in blocking buffer for 1 h). Upon washing three times with a blocking buffer, the cells were incubated with secondary antibodies (goat anti-mouse alexa fluor 488 and goat anti-rabbit alexa fluor 546, Thermo Fisher Scientific, Germany) 1:400 in a blocking buffer for 10 min, followed by three washing steps with a blocking buffer. Images were taken with a Leica SP8X, inverted confocal microscope with 405 nm and pulsed “white light” Laser (470–670 nm) at 40× magnification.

### 4.10. Measurement of SHP-2 Activity

The cells were lysed in a phosphatase lysis buffer (150 mM NaCl, 50 mM Tris-Cl pH 7.4, 5 mM EDTA, Proteinase Inhibitor Cocktail (1:500; Sigma Aldrich), pH 7.35) supplemented with 0.5% NP-40, 0.1% DOC, 0.1% SDS, and 1 mM Na_3_VO_4_ directly before use. Then, 300 µg of total protein was used for the immunoprecipitation of SHP-2 and for measurement of the phosphatase activity, as described previously [35]. 

### 4.11. Statistics

Data were analyzed for statistical differences using Sigma Plot 12.0 (Systat Software, Inpixon, Düsseldorf, Germany). A comparison between two groups was performed using the Mann–Whitney Rank Sum Test for non-paired data. As mean and median differ less than 25%, the mean values are given. For comparison of more than two datasets, one way analysis of variance (ANOVA) followed by the post-hoc tests Student–Newman–Keuls method was used. The results were expressed as mean values ± SEM. Differences were considered significant at *p*-values less than 0.05 (*p* < 0.05).

## Data Availability

The data sets generated during the current study are available from the corresponding author upon reasonable request.
